# Public funding and young children vaccination coverage: Evidence from Socialist-Oriented Market Economy

**DOI:** 10.1186/s13561-024-00569-5

**Published:** 2024-11-20

**Authors:** Tri-Duc Luong, Dao Le-Van

**Affiliations:** International School, Vietnam National University, Hanoi, Vietnam

**Keywords:** Public funding, Vaccination rate, Vietnam, Social objectives, I18, H51, O53

## Abstract

This study presents empirical evidence on the impact of public funding on the vaccination rate of children under one-year-old in Vietnam from 2014 to 2019. The research findings indicate that, *first*, the effect of government funding on the vaccination rate of children is positive after addressing endogeneity, cross-sectional dependence, and heteroscedasticity. *Second*, this impact is more pronounced in underdeveloped regions, particularly those with low female school enrollment rates and underdeveloped infrastructure. This raises a dilemma for Vietnam in pursuing a comprehensive development strategy, as investment in underdeveloped regions yields significantly lower economic returns. Therefore, this study provides further insight into the effectiveness of public funding in pursuing social objectives while initiating discussions regarding policies to achieve multiple goals as the Socialist-Oriented Market Economy reign.

## Introduction

The COVID-19 pandemic significantly tested the medical system’s capacity to endure, treat, and overcome a global health catastrophe. Fortunately, the pandemic has subsided; the World Health Organization announced the end of the “public health emergency” on the 5th of May, 2023. In retrospect, many factors contributed to the global effort, such as international scientific collaboration [25], effective quarantine and tracing policies [6], and increased public awareness initiatives [26]. However, these efforts remained at the surface level and granted no long-term immunization and resistance against future COVID-19 infections. It is worth noting that if health outcomes are considered as a commodity, they exhibit characteristics of asymmetric information, limited competition, extensive externalities, and pervasive uncertainty and risk. As emphasized by Deaton [12], “the great escape” from poverty and suffering in history lies in addressing healthcare challenges. Thus, effective government intervention in national healthcare is regarded as an indispensable and unavoidable solution.

One of the classic examples frequently cited in most textbooks regarding successful government interventions is the provision of vaccines, wherein their positive externalities lead to market failure. On a broader scope, assessing the effectiveness of vaccine service delivery, particularly during crises, can be a crucial clue to evaluating governmental performance [13]. During the COVID-19 pandemic, vaccine deployment has halted the virus’s spread, stopping potential infections and fatalities [27, 80]. To maximize vaccination benefits, however, a nation must have the capacity and facilities to deploy and administer doses to the population effectively. Developed countries such as the USA can quickly deliver shots [68] while less developed nations suffer from shortages to inadequate healthcare infrastructure, impairing their vaccination efficiency [86]. Given the discussion above, researched insights into the significance of public funding towards improvements in health metrics hold large implications on governmental spending policies, especially for low and middle-income countries that have to allocate limited resources.

One intriguing case study in this regard is Vietnam, characterized by two notable features. *First*, Vietnam is transitioning from a centrally planned economy to a socialist-oriented market economy, having significant conflicts between pursuing overall welfare goals (e.g., health outcomes) [54, 82] and efficient economic growth. Thus, examining the government’s expenditure allocation towards achieving social objectives in Vietnam will provide further insights into how those countries can achieve both economic and social objectives. *Second*, Vietnam’s adaptation to COVID-19 has shown an interesting trajectory from outstanding adaptive measures in containing the COVID-19 outbreak (even gaining positive GDP growth in 2020) [43, 73] to a crisis state due to the surge of the pandemic (since April 2021) [33]. It is noteworthy that while the former period recorded success due to rapid vaccination strategies [43, 73], the latter phase resulted in severe economic damage to the population, particularly affecting women, the elderly, and vulnerable individuals in society. Outside of emergency initiatives, Vietnam operates many public health programs in the form of universal health insurance [37], commune-level health directives (Directive no.25 (2023) by the Central Secretariat Committee), and high-quality national hospitals located in Hanoi and Ho Chi Minh City.

Therefore, this study has three contributions as follows. *First*, it offers fresh evidence concerning Vietnam’s situation over a decade before the onset of the COVID-19 pandemic, elucidating why public funding, proxied by the government spending scale on social securities, contributes to an increase in the vaccination rate for children under 1-year-old. Specifically, we endeavor to explicate the mechanisms at play within the context of Vietnam, which, as observed, exhibits notable differences (albeit simpler) compared to more developed nations (e.g., Australia, the United States, or the United Kingdom). *Second*, the study confirms empirical findings on heterogeneous impacts of the current nexus given various contextual conditions, including (i) provinces with well-developed infrastructure conditions compared to those with poor conditions and (ii) provinces facilitating educational opportunities for girls versus those that do not. Consequently, we propose policies regarding whether the government should allocate funds to which areas to achieve optimal outcomes in health. *Third*, technically, this work delves into the causal impact of public funding on vaccination rather than the correlation relationship. To achieve this, we employ a generalized method of moments (GMM) estimator by introducing new external instruments, which are derived from an understanding of the policy allocation system in Vietnam. Besides, the current design, utilizing the unit of analysis as the 63 provinces/cities of Vietnam, enables the fulfillment of the independent and identically distributed conditions of the observations in the study sample (see also [34–36]).

The remaining sections of this study are outlined as follows. Sect. "Literature review" presents the body of knowledge showing the nexus between public funding and health outcomes, emphasizing contextual factors. Sect. "Methodology and data" elucidates the research methodology and data, while Sect. "Results" provides the study results. Sect. "Discussion and policy implication" offers discussions and proposes some relevant policies in Vietnam.

## Literature review

Health outcomes and public funding have been studied extensively at the national and international levels [4, 50, 55]. Anwar et al. [4] assessed 38 Organisation for Economic Co-operation and Development countries from 1996 to 2020 and found decreasing infant mortality and increasing life expectancy when gross domestic product (GDP), healthcare spending, and environmental factors improved. Onofrei et al. [50] arrived at similar conclusions about public funding for health but remarked that “socio-economic vulnerabilities” and stable political climates are also important in determining health outcomes. Similarly, studies examining health budget cuts after COVID-19 reported decreased healthcare quality at the local [64] and national level [28].

However, other studies provide conflicting views. Filmer and Pritchett [15] stated *“The small impact of health spending translates into very low explanatory power”*(p. 1315); Kim and Moody [29] suggested that public health funding (PHF) plays a small, secondary role to other socioeconomic resources such as income. A more recent study from Oman also claimed no significant effect from health funding on non-communicable diseases but was significant for other dependent variables such as life expectancy [2]. We are not aware of any studies stating a negative relationship between public funding and health outcomes.

Explanations regarding the mechanism of public spending increase are still underdeveloped in current public health literature and can only be derived from theoretical studies and frameworks. The mechanism from which the effect of increased spending is derived can be viewed from a functional perspective or an economic perspective. The functional perspective was presented by Kutzin [30], in which the public health financing system consists of four stages: collection of funds, pooling of funds, purchasing of services, and provision of services. The collection and pooling of funds is implied to be at the national level, and thus an increase in spending will flow downstream, affecting service purchase and provision. The economic view (Grossman model) treats health as an accumulated capital (consisting of innate and invested health components) that depreciates over time due to aging [21]. While the innate component is mostly static, the invested component is dynamic, in which the optimum quantity of health units is determined by wage, medical service cost, age, initial health, and education. An increase in health funding likely modifies medical service costs, increasing optimum health units (i.e. an increase in health outcomes). The health stock in Grossman’s model is considered within an individual utility function, with a clear trade-off between investing into health or consumption.

It is interesting to note that outside of the easily conceivable variables in PHF models, there exists overarching yet subtle differences between healthcare systems that may bias effectiveness in certain areas. Ivanková et al. [22] assessed three different types of healthcare systems across five areas and found that each system excelled and fell behind in different areas. The effect of PHF in each of these systems will be biased towards select specialties. Additionally, each country’s political and financial stability is a prerequisite for a consistent effect of PHF [17, 24]. Finally, there are critical but hard-to-quantify factors such as climate and climate change tendencies [41] and genetic dispositions [23].

While surveying countries and regions can cover a large part of the population and utilize large datasets, there are variables between countries that create difficulties in generalizing and applying a model. In the specific case of healthcare funding, important decisions regarding resource allocation must be made, which can not be satisfactorily answered using a blanket national model. Sub-national methodologies focus on comparing data between the sub-divisions of a nation to draw conclusions, which has strengths in increasing observation size and similarity [63]. The sub-national perspective has been applied in many studies, for example, policy [85], foreign direct investment [7], and health [51].

Studies with sub-national research design on health funding and outcomes have recently been conducted using data from US states [9]. In Cardona et al. [9, 14], the effect of various socio-economic variables on county health outcomes was examined. It was found that spending on infrastructure generated more effect in urban counties, while investments in social services (including health) better benefitted rural areas. Lamba et al. [31] explored the effect of local spending on COVID-19 response, while Vilda et al. [79] assessed state and local spending on pregnancy mortality. Overall, the sub-national level is a new and rising approach used to explore more detailed and intricate relationships that higher levels of data (national, cross-national) might omit.

Specific literature on the relationship between government health spending and vaccination rates reported positive effects [16, 46]. In addition, many studies cite factors directly related to government health spending as significant predictors of vaccination rates such as natal care facilities [1, 65, 66], vaccine stock and shortages [58]. Literature pertaining explicitly to the relationship between public funding and vaccines mainly focused on low and middle-income countries, while studies focusing on developed countries were rarely encountered.

To the best of our knowledge, there are no studies connecting public spending and vaccination outcomes in Vietnam. Previous studies published in Vietnamese have mainly focused on calculating the cost and efficiency of vaccine programs in Vietnam [32, 52] instead of developing a quantitative model. This study is the first to closely examine the effects of public funding on vaccination rates in Vietnam, a developing country with a unique socialist-oriented economic system. In addition, the study will attempt to provide a plausible mechanism for the observed effect of social spending increase, an area still lacking in present empirical studies.

## Methodology and data

### Theory and basic setup

We follow the assumptions and framework described in the Grossman model of health [21] as discussed in Sect. "Literature review". Health is treated as an accumulated but depreciating capital, with the optimum health status modified by wage, education, cost, initial health, and age. In theory, an increase in public spending will subsidize the cost and increase the optimum health units synonymous with better outcomes. However, the health stock is nested within a utility function that allows for calculations on equilibrium and related economic points of interest.

From an economic perspective, vaccines—considered a public good—are expected to be efficiently provided through government expenditure, as anticipated by Public Goods Provision Theory [48, 59, 69]. It underscores the critical role of well-planned public spending in fostering economic growth and welfare by facilitating collective action and coordinating resources effectively. In the battle against COVID-19, the theory highlights the necessity of collective action to provide health goods and services that benefit society, irrespective of individual contributions, as follows. *First*, the notion of non-excludability, a key characteristic of public goods, emphasizes that it becomes practically unfeasible to exclude individuals from its benefits once a vaccine becomes available [45]. *Second*, considering the presence of positive externalities, widespread vaccination not only offers protection to the vaccinated individual but also fosters herd immunity, thereby safeguarding the entire population, including those unable to receive the vaccine due to medical reasons. Consequently, the private sector may consistently provide below the necessary level that society requires [43, 73].

*Third*, the free-rider dilemma, where individuals may refrain from contributing to the provision of public goods while still benefiting from them, necessitates the implementation of mechanisms to address vaccine hesitancy and ensure compliance [61]. Drawing from insights in behavioral economics and social psychology, interventions, thus, such as targeted messaging, incentives, and community engagement, can effectively counter misinformation, instill trust in vaccines, and promote vaccination as a social norm [8]. Said utility function cannot be used to estimate marginal effects on vaccination rates, and the relevant variables (health, wage, education, cost, initial health, and age) must be adapted to a stand-alone econometric model that fits our current research data and objectives. Thus, our model is as follows:

1 $${Y}_{i,t}= {\alpha }_{i}+{{\beta }_{1}{Y}_{i,t-1}+ \beta }_{2}{GEOSS}_{i,t-s}+Z\theta +\lambda t+{\varepsilon }_{i,t}$$

where ${Y}_{i,t}$​ denotes the vaccination rate among children in province *i* during year *t*. An essential point of contention pertains to the presence of auto-correlation in the response variable, which arises from the periodic expenditure plans of local governments typically scheduled every five years as opposed to random variations, often made in accordance with federal expenditure plans [72]. Consequently, controlling for the lag of the dependent variable allows for an assessment of any residual statistical relationship between these variables and the response after the mitigation of auto-correlation effects (${\beta }_{2}$​). *GEOSS* represents government expenditure on social securities, a proxy for public spending, with *s* denoting the lag in the impact on health outcomes. Previous studies have often established a lag of 1, which can be attributed to the transition of public funds towards local healthcare activities [56]. Specifically, Reibling [56] calculated mean lags of public national healthcare expenditure to be 2 years, making a 1-year lag for local expenditures highly reasonable since the scale is smaller.

*Z* signifies the vector of control variables, among which the expenditure on social welfare programs in Vietnamese provinces significantly influences inter-provincial population migration, particularly within the Southeastern region to Binh Duong and Ho Chi Minh City. The substantial population movements illustrate this phenomenon observed during COVID-19 [18]. Population dynamics evidently affect vaccination rates. Another crucial factor necessitating control is literacy. Educational attainment directly impacts children’s vaccination by fostering awareness of vaccines’ role in preventing severe illnesses such as hepatitis B, polio, rabies, and measles [42]. It should be noted that tropical diseases exhibit significantly graver implications compared to temperate regions, a discernment relatively well-acknowledged within these areas [84].

Notably, increased expenditure on social welfare partly enhances literacy through various means, particularly by disseminating information regarding diseases preventable through vaccination. Other influential factors in the model may include (i) the development of the private sector and (ii) per capita income growth. Specifically, private sector development augments government tax revenues, expenditure, and the quality of local healthcare services. Per capita income growth improves children’s access to healthcare services (especially vaccination) early in life, increasing tax revenues from personal income taxes or corporate income sources. Parameters *α*, *λ*, and *ε* represent country-fixed effects, time-fixed-effects, and error terms, respectively. While country fixed-effects control for time-invariant unobservable factors (such as cultural, historical, and ethnic factors), time fixed-effects regulate time trends.

Furthermore, to examine the heterogeneous effects of $GEOSS$ on vaccination rates across different contexts, such as variations in institutional quality or regions exhibiting greater gender (ethnic) inequality, we investigate the interactive effects using the following model.

2 $${Y}_{i,t}= {\alpha }_{i}+{{\beta }_{1}{Y}_{i,t-1}+ \beta }_{2}{GEOSS}_{i,t-s}+{\beta }_{3}{GEOSS}_{i,t-s}\times \overline{M }+Z\theta +\lambda t+{\varepsilon }_{i,t}$$

where $\overline{\text{M} }$ represents the average value of the moderating variables over the entire period. In this case, ${\beta }_{3}$​ reflects the heterogeneous effects of contextual factors.

### Endogeneity

The research design outlined above, with its intricate interplay of factors, may raise endogeneity concerns, stemming from the complex issues of simultaneity and confounding. *First*, simultaneity occurs when provinces achieving vaccination targets set for each term (5 years) receive more budgetary resources for local development, subsequently increasing expenditure on social welfare. *Second*, more common criticism may arise from confounding issues, which suggest that other factors influence concurrent improvements or declines in childhood vaccination rates and social welfare expenditure. For example, the ideological shift known as “Doi Moi,” which facilitated access to the domestic free-market economy in the medical sector and contemporary fiscal policies, led to the rapid expansion of private vaccination services and changes in the structure of social welfare budgets. Ordinary least squares (OLS) with fixed effects may exhibit bias and inconsistency in such scenarios.

To address the endogeneity problem, we focus on analyzing changes in exogenous factors (referred to as instrumental variables) that solely influence vaccination rates through variations in local budget allocation: specifically, the design incorporates two sources of budgeting: (i) balanced tax revenues from the central government and (ii) targeted tax revenues. In Vietnam, fiscal decentralization under the integrated budget system prevails, where the state budget is managed and determined solely by the Central Government [47]. The structure of local budget expenditure comprises approximately 70% allocated to development and recurrent spending, with the remaining 30% allocated to various other expenses (e.g., supplementary financial reserve fund, carry-over spending, and transfers to the following year’s budget), wherein expenditure for social securities—a main focus of this study—lies within the recurrent expenditure category, defined as “*obligatory expenditures of the state budget on maintenance of operation of the State apparatus, political organizations, socio-political organizations, support for operation of other organizations, and performance of regular tasks of the State in terms of socio-economic development and assurance of national defense and security*” (Article 4, Clause 6, Law on the State Budget, The National Assembly, 2015, p. 1) [75].

On average, provinces allocate approximately 8% of the total budget to this expenditure, contingent upon socio-economic development conditions and objectives assigned by the central government. However, funding for this aspect can be supplemented through other revenue sources, namely (i) balanced tax sources from the central government and (ii) targeted tax revenues. *First*, balanced tax sources from the central government are typically determined through negotiations between local authorities and the central government to achieve socio-economic objectives (e.g., within the framework of 5 or 10-year plans). Also, a local area or economic region may increase balanced tax if its socio-economic development has been weak for an extended period (e.g., the Mekong Delta region), prompting the central government to allocate more resources to ensure social equity (Vietnam Chamber of Commerce and Industry [VCCI] & Fulbright University Vietnam [FUV], [71]).

*Second*, targeted tax revenues are specific revenue sources allowed by the central government to be collected (without division) by localities to serve socio-economic development tasks (commonly known as “special mechanisms,” examples include Resolution No. 119/2020/QH14 for Da Nang and Resolution No. 38/2021/QH15 for Hue). The attainment of these special mechanisms depends on various factors, such as the scale and pace of local development and the negotiating capacity of local authorities with the central government. Thus, it is reasonable to assume that the vaccination rate has not influenced decisions to improve these two budget sources in the past 1–3 years. Therefore, examining the impact of GEOSS on children under one year of age who are fully vaccinated through the channels of (i) balanced tax sources from the central government and (ii) targeted tax revenues allows for precisely determining causal relationships.

Notably, when employing the lag of the dependent variable control model, the general method of moments (GMM) estimator offers a superior estimate compared to the two-stage least square (2SLS) approach using lagged instrumental variables [57, 81]. However, due to its complexity, GMM may induce spurious results with weak instrumental variables and detection difficulty. The quest for a good (i.e., exogenous and pertinent) instrumental variable remains an ongoing challenge in empirical socio-economic research, requiring continued contributions from subsequent studies. By rule of thumb, coefficients derived from the two-stage least squares method ought not to exceed three times the magnitude of those obtained from the ordinary least squares and fixed-effects models. Given the above discussion, in our case, technical tests will be presented alongside the aforementioned theoretical rationale to ensure the validity of the instrumental variables, thereby ensuring coefficient consistency. The estimated values derived from the initial regression of Eq. (1) are detailed below:

3 $$GEOS{S}_{it} = f(I{V}_{i,t-1}, GEOS{S}_{i,t-1}, Z)$$

### Data

We utilize panel data encompassing 63 provinces/regions of Vietnam from 2014 to 2019. To search relevant literature for a similar theoretical basis, we must first make some crucial statements regarding Vietnam as an ideal case for research design; it is imperative to establish Vietnam as an ideal case for research design. First and foremost, Vietnam is a developing country with low-middle to middle income and a healthy, young, and increasingly educated labor force, while its economic performance is stable and is projected to continue [83]. Furthermore, the political landscape in Vietnam is characterized by stability, with the government fostering an open market environment [19]. Regarding research design, it is noteworthy that (i) Vietnamese provinces/regions share a common cultural heritage and longstanding history, while (ii) maintaining relative policy independence allows for practical examination of causal relationships. This unit of analysis has been leveraged in numerous recent studies [34–36].

The study relies on three primary data sources: the general statistical yearbooks (GSO), the provincial competitiveness index (PCI), and data from the Ministry of Finance. *First*, GSO provides data on the vaccination rate of children under one year across all 63 provinces/regions nationwide and GEOSS. Utilizing these two variables with the same source allows us to mitigate statistical errors. *Second*, PCI (accessible at https://pcivietnam.vn/) offers insights into the institutional status of provinces/regions from a business perspective, enabling us to examine hypotheses regarding heterogeneous effects within inefficient local governance. Third*, *the Ministry of Finance provides data on balanced tax sources from the central government and targeted tax revenues. As described, this dataset facilitates research design to address endogeneity issues and spans 2014 to 2019. Additional control data are collected and detailed in Table 1, with varying data availability across variables. Furthermore, data collection predates the COVID-19 pandemic (in 2020) to minimize any alterations in vaccination behavior among the populace.

**Table 1:** Descriptive statistics

	Sources	Unit	Obs	MEAN	SD	MIN	MAX
Dependent variables							
Children fully vaccinated	GSO	[0,1]	504	0.956	0.044	0.640	1.000
Independent variables							
GEOSS over GDP	GSO	(%)	739	8.140	5.526	0.006	34.944
GEOSS on logarithm	GSO	Bill. VND	731	5.560	0.986	2.460	8.000
Control variables							
Population on logarithm	GSO	Thous. People	743	7.066	0.583	5.592	9.109
Literacy	GSO	%	756	92.631	7.039	59.200	99.200
Private sector labor share	Le-Van and Tran [34]	%	716	88.327	10.305	29.790	99.281
GRDP per capita on logarithm	GSO	mill. VND	743	3.282	0.566	2.150	5.466
Variables for mechanism tests							
Nurse numbers on logarithm	GSO	1 person	630	6.883	0.629	4.533	9.558
Midwives number on logarithm	GSO	1 person	630	5.896	0.500	4.543	7.969
Nurse over hospital on logarithm	GSO	person/1 hospital	630	4.223	0.411	2.037	5.646
Nurse over medical station on logarithm	GSO	person/1 station	629	1.846	0.496	-0.209	3.783
Midwives over hospital on logarithm	GSO	person/1 hospital	630	3.235	0.356	2.150	4.032
Midwives over medical station on logarithm	GSO	person/1 station	629	0.856	0.462	-0.481	2.204
Moderate variables (Female students, Institutional quality, and Infrastructures							
Female student on logarithm	GSO	1 person	756	11.528	0.534	10.058	13.466
Preprimary female student on logarithm	GSO	1 person	756	10.796	0.527	9.268	12.785
Secondary female student on logarithm	GSO	1 person	756	10.432	0.537	8.940	12.323
High school female student on logarithm	GSO	1 person	756	9.794	0.632	7.881	11.721
Business support services	PCI^a^	[0,10]	630	5.449	1.177	1.750	8.750
Law & Order	PCI	[0,10]	630	5.551	1.101	2.000	7.990
Class numbers on logarithm	GSO	1 class	756	8.843	0.453	7.732	10.521
School numbers on logarithm	GSO	1 school	693	6.019	0.414	5.142	7.383
Hospital numbers on logarithm	GSO	1 hospital	630	0.996	0.130	0.476	1.397
Library number on logarithm	GSO	1 library	567	2.643	0.601	1.609	5.710
Library book numbers on logarithm	GSO	1 book	554	5.607	0.652	2.797	8.202
Magazine office on logarithm	GSO	1 office	630	1.471	0.798	0.693	6.480
External instrumental variable							
Balance tax sources from the central government and targeted tax revenues on logarithm	Ministry of Finance	Mill. VND	630	16.310	0.557	15.185	18.857

Source: Authors. Vaccination data is available from 2010 to 2019, excluding 2011 and 2012, which gives a maximum of 504 observations. Other datasets begin in 2008, allowing for up to 756 observations in total

^a^https://pcivietnam.vn/

## Results

### Background of the study

**Fig. 1:**
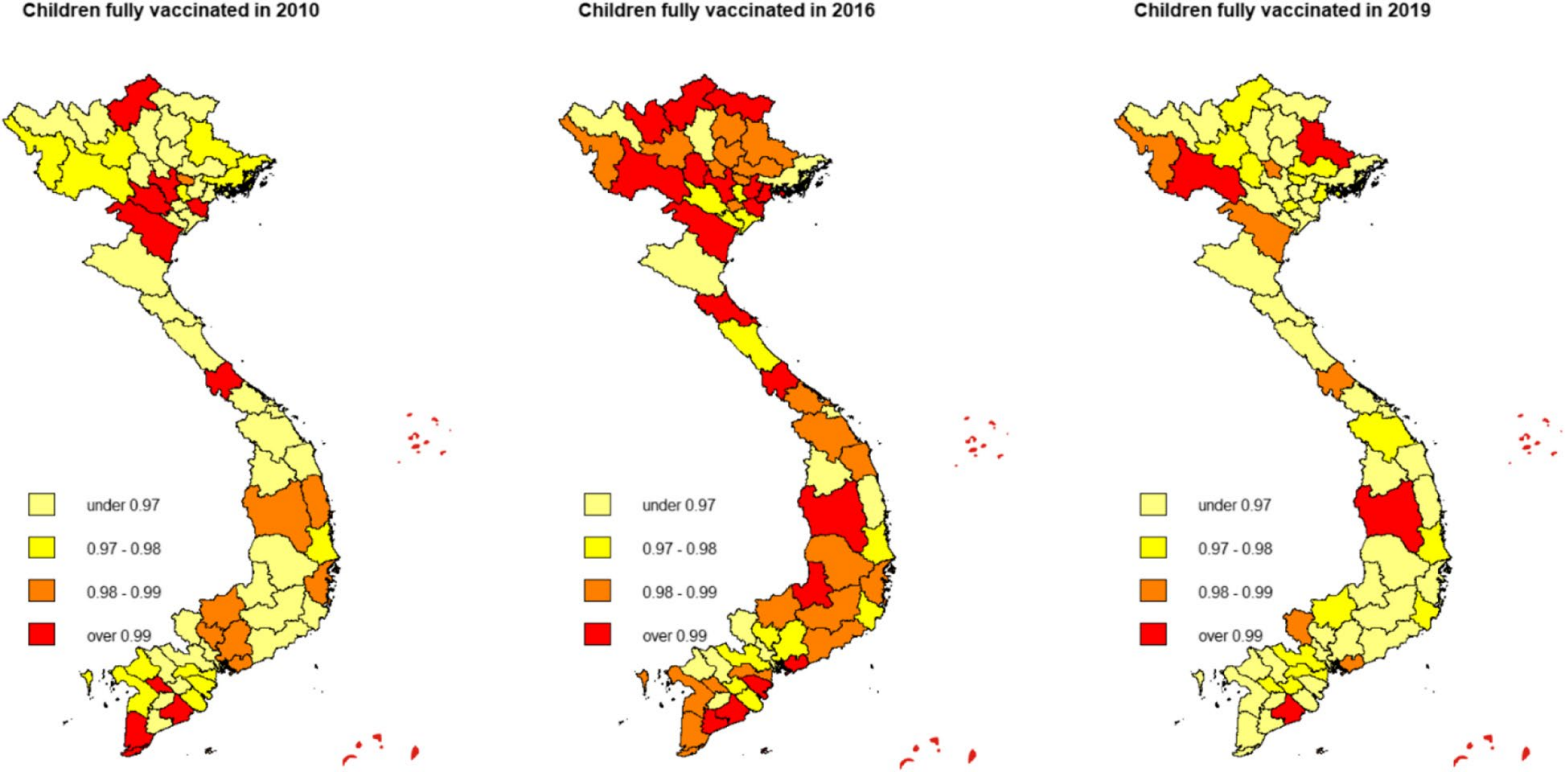
Vaccine vaccination rate of children under 1 year old in Vietnam, 2010–2019. Source: Authors

**Fig. 2:**
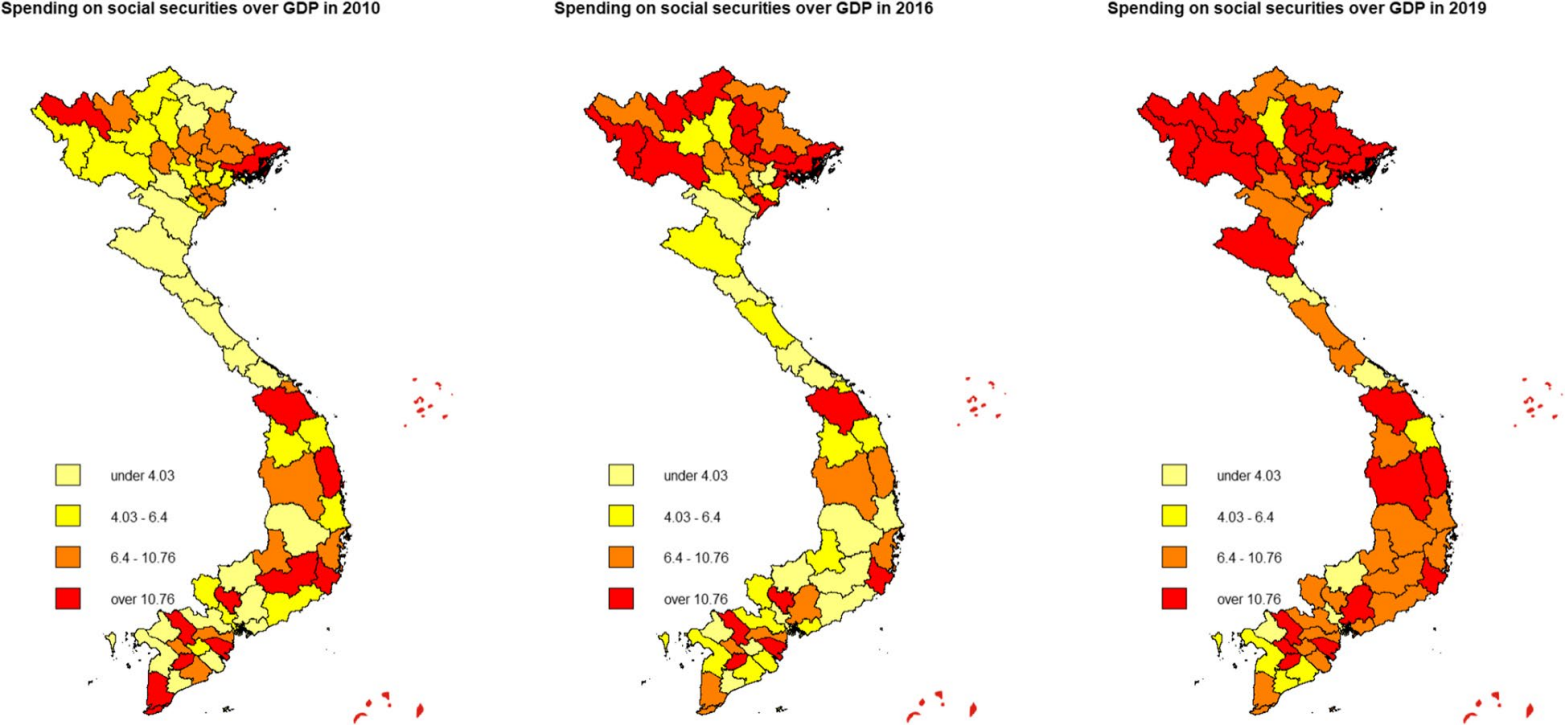
Government spending on social securities in Vietnam, 2010–2019. Source: Authors

The overall vaccination rate in Vietnam was higher in 2016 compared to 2010 levels, but the 2019 data suggests a heterogeneous decrease in nationwide coverage (Fig. 1). On the contrary, GEOSS increased uniformly throughout the 2010–2019 period (Fig. 2). Both maps are consistent for the 2010–2016 period but inconsistent for the 2016–2019 period. This divergence in 2016–2019 is explained by the introduction of a new mandated vaccine for children under one-year-old in 2018 by the Vietnamese Ministry of Health. In 2018, a new pentavalent (5-in-1) vaccine named ComBE Five (India) was introduced, replacing the already-in-use Quinvaxem (Korea). By December 2018, the ministry authorized the nationwide deployment of the CombeFive vaccine [70]. However, the deployment was fraught with vaccine shortage issues as reported by official Vietnam state media (Vietnam Television [VTV] [77]). In addition, fatalities and hospitalizations after vaccination caused parents to refuse further doses (Vietnam Television [VTV] [78]). Consequently, vaccination rates dropped significantly in 2019. We verified this argument by running a simple univariate regression (see Appendix [1] for more details). The fallout from these events forced the government to increase safety measures, improve vaccine acquisition, and restore public trust, manifesting as an increase in public spending indicated by Fig. 2. In the context of our econometric model, we thus controlled for time-fixed-effects ($\lambda$).

**Fig. 3:**
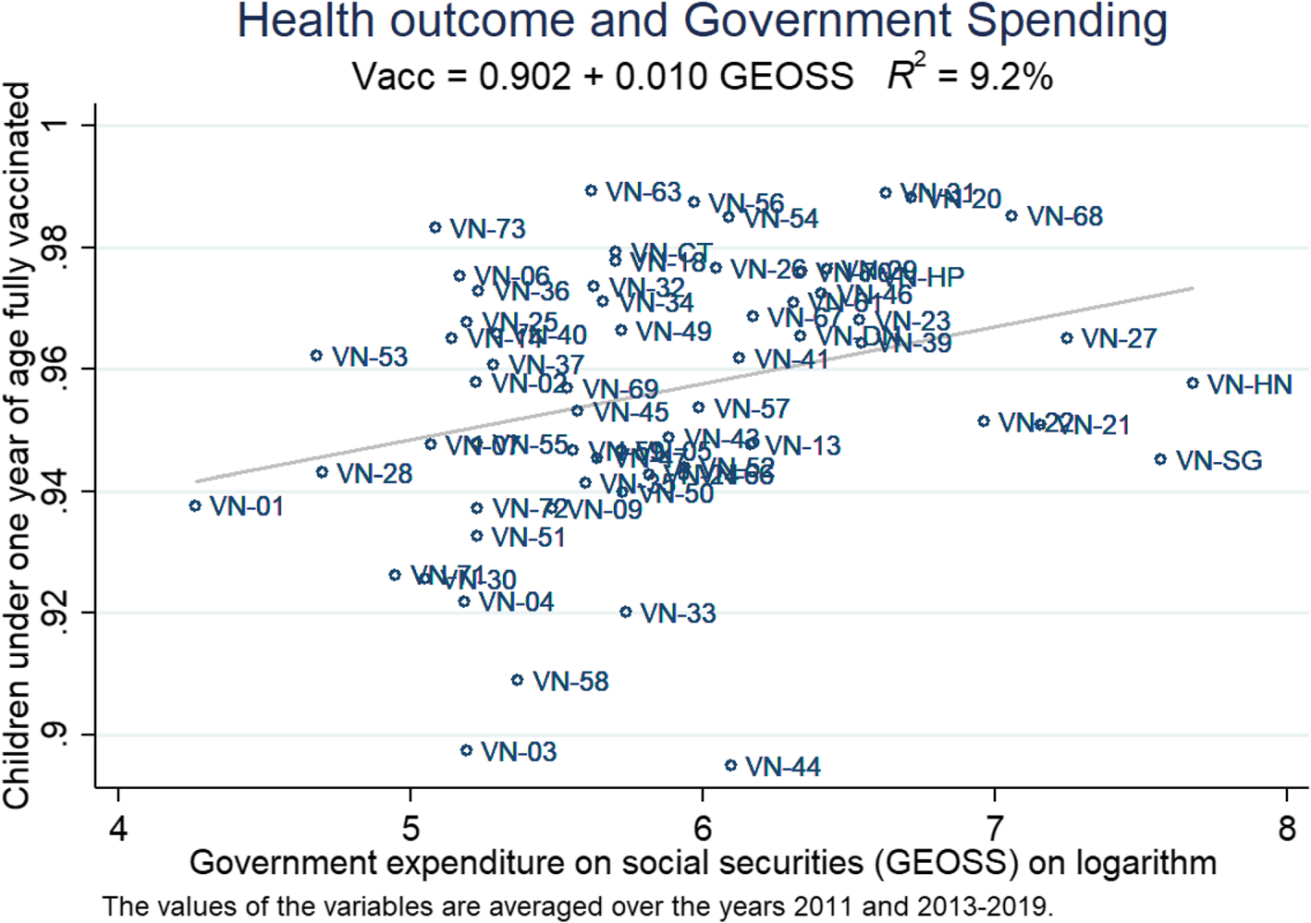
Health outcome and government spending on social securities. Sources: Authors’ own work. Notes: The data is averaged, including the years 2010 and 2013 to 2019 (as data for 2011 and 2012 is inaccessible). The names of the provinces are abbreviated according to Standard ISO 3166 (see https://www.iso.org/obp/ui/#iso:code:3166:VN for more details)

Figure 3 demonstrates a positive correlation between the averaged values of GEOSS and the vaccination rate. The large majority of provinces received funding levels from 5 to 6, while 4 provinces received less than 5, and 6 provinces received higher than 6. The two provinces with the highest funding were Hanoi (VN-HN) and Ho Chi Minh City (VN-SG), while the least funded province was Lai Chau (VN-01), a highly rural and remote province in the Northwestern highlands of Vietnam. 94% to 98% appears to be the standard range of vaccination coverage, with about one-third placing outside this range. Interestingly, despite heavy funding, Hanoi and Ho Chi Minh City only achieved approximately 95% coverage, while Lai Chau stands at nearly 94%.

To better understand our proposed mechanism for GEOSS, several key information regarding the Vietnamese system and vaccination procedures must be introduced. *First*, Vietnamese law allows for vaccination operations at all registered and certified facilities (public and private). Nurses and midwives are allowed to administer vaccines, but the law requires that at least 1 doctor assistant or higher must be on-site (refer to [20]). *Second*, most vaccinations occur at local-level facilities, which feature a large percentage of nurses and midwives to doctors. Excluding higher income families that can afford private vaccinations, most Vietnamese choose to vaccinate locally. Usually, a vaccination operation is organized monthly by the health authorities at local health stations. Children and parents can enter without an appointment, receive a quick vaccination, and return home on the same day. The no-appointment system provides the highest convenience for patients but results in high workloads for the understaffed health stations.

**Fig. 4:**
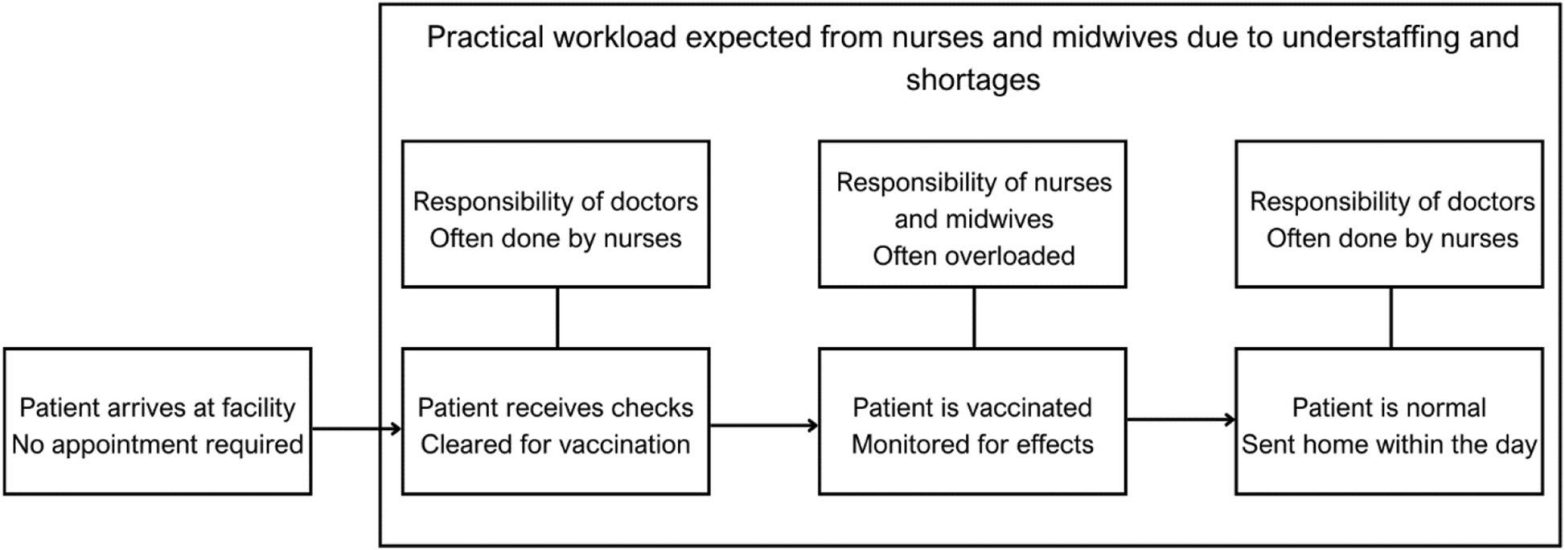
Condensed Vietnamese vaccination procedure and staffing challenges. Source: Authors’ own work

Finally, the standard vaccination procedure begins with doctors or doctor assistants examining the patient before injection. Nurses and midwives then administer the dose, and the patient is monitored before being discharged. In practice, most of the vaccination procedures are handled by nurses and midwives (for newborns taking hepatitis B and tuberculosis shots) with minimal involvement from doctors or doctor assistants due to a chronic shortage of local-level medical personnel. Figure 4 illustrates a simplified way of vaccinating children in Vietnam.

### Main results

Table 2 shows the impact of $GEOSS$ with various lag lengths in a fixed-effects model, controlling for temporal and provincial variables. Utilizing a lagged variable of $t+1$, GEOSS exhibited no significant effect on the dependent variable, as we expected. The current ($t$) and one-year lagged effects ($t-1$) demonstrate increasing positive impacts and statistical significance levels (0.008 at $p<0.1$ for $t$, 0.011 at $p<0.05$ for $t-1$). Thus, using the GEOSS variable with a 1-year lag is optimal.

**Table 2:** The impact of GEOSS on children’s vaccination rates A

Dependent variable:	Children under one year of age fully vaccinated			
Estimator	FEM	FEM	FEM	FEM
Columns	(1)	(2)	(3)	(4)
Children fully vaccinated_t-1_	-0.035	-0.035	-0.030	-0.034
	(0.038)	(0.041)	(0.041)	(0.038)
Government expenditure on social securities_t+1_ (GEOSS_t+1_)	0.002			0.003
	(0.004)			(0.004)
Government expenditure on social securities_t_ (GEOSS_t_)		0.008*		0.003
		(0.004)		(0.004)
Government expenditure on social securities_t-1_ (GEOSS_t-1_)			0.011**	0.008*
			(0.004)	(0.004)
Time Fes	Yes	Yes	Yes	Yes
Province Fes	Yes	Yes	Yes	Yes
Observations	315	378	378	315
R-squared	0.538	0.541	0.544	0.545

Source: Authors. Columns [1] and [4] include the years 2011 and 2014–2018, while columns [2] and [3] cover the years 2014–2019. Standard errors are indicated in parentheses with significance levels: *** for *p* < 0.01, ** for *p* < 0.05, * for *p* < 0.1

Table 3 presents the effect of $GEOSS$ on the vaccination rate of children under one year old, revealing consistent results after controlling for dynamics impact (Column [1]) and mitigating omitted variable bias by including other control variables (Columns [2]-[6]). Under the ordinary least squares model, $GEOS{S}_{t-1}$ exhibited a standalone coefficient of 0.007 at the 0.01 significance level (Column [1]), while the estimated coefficient decreased to 0.004 after controlling for the presence of auto-correlation in the response variable (Column [2]). Controlling for population (negative correlation with vaccination rates) increased the coefficient from 0.007 to 0.009 (Column [3]), indicating the increased impact of GEOSS functions through the mechanism of population growth in the considered area. Given the shutting down literacy mechanism (positive correlation with vaccination rates), the coefficient marginally decreased to 0.007 (Column [4]). Indeed, increasing expenditure on social welfare enhances public awareness (through campaigns and educational subsidies), thereby increasing vaccination rates.

**Table 3:** The impact of GEOSS on children’s vaccination rates B

Dependent variable:	Children under one year of age fully vaccinated					
Estimators	OLS	OLS	OLS	OLS	OLS	FEM
columns	(1)	(2)	(3)	(4)	(5)	(6)
Government expenditure on social securities_t-1_	0.007***	0.004**	0.009***	0.007***	0.007***	0.009**
	(0.002)	(0.002)	(0.002)	(0.002)	(0.002)	(0.004)
Children fully vaccinated_t-1_		0.241***	0.235***	0.184***	0.167***	-0.037
		(0.060)	(0.059)	(0.055)	(0.053)	(0.043)
Population on logarithm			-0.009**	-0.014***	-0.014***	0.016
			(0.004)	(0.004)	(0.003)	(0.028)
Literacy				0.001***	0.001***	0.000
				(0.000)	(0.000)	(0.002)
Private sector labor share					0.000***	0.001
					(0.000)	(0.001)
GRDP per capita on logarithm					-0.000	0.005
					(0.003)	(0.026)
Time Fes	Yes	Yes	Yes	Yes	Yes	Yes
Province Fes	No	No	No	No	No	Yes
Observations	493	378	378	378	378	378
R-squared	0.251	0.233	0.247	0.302	0.312	0.558

Source: Authors. Column [1] includes the years 2010 and 2013–2019, while columns [2]-[6] cover the years 2014–2019. Standard errors are indicated in parentheses with significance levels: *** for *p* < 0.01, ** for *p* < 0.05, * for *p* < 0.1

In Column [5], we control for factors that may produce cofounder effects, such as private sector labor share and gross regional domestic product, yielding a robust impact with an estimated coefficient of 0.007. Finally, after controlling for time-invariant unobservable factors, the coefficient for GEOSS remains at 0.009 with the same significance level as the ordinary least squares model, indicating a positive effect of GEOSS on children’s vaccination rates. Only slight changes in the coefficient were observed with the addition of control variables. Results from the fixed-effects model align with Table 2 and previous studies conducted for sub-Saharan Africa [10], European Union developing nations [50], and South East Asia [62]. Some recent evidence from the United States [14] reported no significant impact but the lack of endogeneity controls in the study may affect the results.

With the context of Vietnam in mind (see Fig. 4), it is reasonable to conclude that nurses and midwives are highly correlated with vaccination quality. Thus, Table 4 illustrates the effect of variables on quantity and quality indicators related to nurses and midwives. Only $GEOSS$ exhibited consistently positive and significant effects across quantity and quality indicators with temporal and provincial factors controlled. Private sector labor share shows a smaller effect on number of nurses (0.007 at *p* < 0.01) and can likely be attributed to the recent development of private hospital chains in Vietnam.

**Table 4:** Mechanism

Dependent variables	Quantity:		Quality:			
	Nurse on logarithm	Midwives on logarithm	Nurse over hospital on logarithm	Nurse over medical station on logarithm	Midwives over hospital on logarithm	Midwives over medical station on logarithm
Estimators	OLS	OLS	OLS	OLS	OLS	OLS
Columns	(1)	(2)	(3)	(4)	(5)	(6)
Government expenditure on social securities_t-1_	0.046***	0.029***	0.055***	0.048***	0.038***	0.031***
	(0.017)	(0.010)	(0.018)	(0.017)	(0.012)	(0.010)
Population on logarithm	0.020	-0.009	-0.117	-0.030	-0.146	-0.059
	(0.149)	(0.084)	(0.158)	(0.151)	(0.101)	(0.083)
Literacy	-0.013*	0.007*	-0.016**	-0.014**	0.003	0.005
	(0.007)	(0.004)	(0.007)	(0.007)	(0.004)	(0.004)
Private sector labor share	0.007***	0.002	0.008***	0.008***	0.002	0.002
	(0.002)	(0.001)	(0.002)	(0.002)	(0.002)	(0.001)
GRDP per capita on logarithm	-0.022	-0.070	-0.131	-0.062	-0.180**	-0.112
	(0.125)	(0.070)	(0.132)	(0.126)	(0.084)	(0.070)
Time Fes	Yes	Yes	Yes	Yes	Yes	Yes
Province Fes	Yes	Yes	Yes	Yes	Yes	Yes
Observations	534	534	534	533	534	533
R-squared	0.930	0.965	0.821	0.884	0.905	0.960

Source: Authors. Columns [1]-[6] cover data from 2011 to 2019, while some missing values result in a maximum of 534 final observations. Standard errors are indicated in parentheses with significance levels: *** for *p* < 0.01, ** for *p* < 0.05, * for *p* < 0.1

From this observation and adopting McKinlay’s model for health promotion in which public spending can be categorized as “*upstream*” interventions [44], we propose a three-stage mechanism to account for the lagged $GEOS{S}_{t-1}$ variable and the fact that decree implementation and subsequent downstream effects take time to materialize: (i) first stage $GEOSS$ is increased through governmental decisions and decrees; (ii) second stage: The increased $GEOSS$ funding is distributed to invest in more local healthcare stations as well as improving benefits to attract higher quality applicants. Investing in more stations and better benefits increases and quantity and quality of healthcare workers; and (iii) third stage: Increased quantity and quality of healthcare workers (specifically nurses and midwives) translates to higher vaccination effectiveness and coverage. The findings align with the systematic review framework outlined in Phillips et al. [53] and Davies et al. [11], stating healthcare facilities and workforce as a determinant of vaccine coverage, especially in transitioning countries.

### Heterogeneous effects and robustness checks

The study was conceptualized with data at the sub-national scale. We sought to not only clarify the generalized effect of $GEOSS$ on children's vaccination rates, but to also evaluate said effect in the context of regional/provincial differences. This is done to more accurately inform future research and policies to target areas that generate the most benefit per unit of public spending.

The selection of moderating variables primarily relies on our observations in the context of Vietnam to determine whether investing an additional dollar in a particular area would yield the most favorable health outcomes. Specifically, one issue is the persistent gender inequality in education access in Vietnam, particularly in the northern mountainous regions (PANEL A). Indeed, early marriage hinders girls’ access to education in the mountainous regions, resulting in less knowledge of childcare [49, 87]. An essential question arises: Should an additional dollar be invested in areas where girls already have good access to education or where girls have limited access to education? Similar discussions are observed and established between regions with (i) well-established systems versus those with underdeveloped systems (PANEL A) and (ii) good infrastructure versus inadequate infrastructure (PANEL B).

The condensed results testing the interaction between $GEOSS$ and the moderating variables are shown in Table 5 (full table available in Appendix [2]). All the moderating variables were taken from averaged provincial data and are characteristic of a specific province. The interaction term between $GEOSS$ and the moderating variables have negative coefficients ranging from -0.013 (number of magazine offices) to -0.071 (number of hospitals) and are all significant at the 5% level. The only exception is the number of female high school students. This is because high school students, in general, have more mobility than preprimary and secondary female students. A secondary female student in Hanoi could have transferred to Ho Chi Minh City for high school, making the statistic less reflective of unique provincial parameters. The result in Table 5 implies diminished benefit from funding for child vaccination coverage when spent on provinces with a high number of female students, institutional quality, and infrastructure (i.e., more developed provinces). Conversely, additional benefits are to be gained if funding is directed at regions with low female students, poor institutional quality, and bad infrastructure (i.e., underdeveloped provinces).

**Table 5:** Heterogeneous effects

PANEL A: Female students & Institutional quality						
Dependent variables: Children under one year of age fully vaccinated						
$Moderate$	Female students				Institutional quality	
	Female student	Preprimary female student	Secondary female student	High school female student	Business support services	Law & Order
Columns	(1)	(2)	(3)	(4)	(5)	(6)
GEOSS_t-1_	0.258***	0.283***	0.222***	0.118*	0.127***	0.118**
	(0.093)	(0.093)	(0.084)	(0.061)	(0.046)	(0.046)
GEOSS_t-1_ $\times \overline{Moderate }$	-0.022***	-0.026***	-0.021**	-0.011*	-0.022***	-0.019**
	(0.008)	(0.009)	(0.008)	(0.006)	(0.009)	(0.008)
Dynamics	Yes	Yes	Yes	Yes	Yes	Yes
Controls	Yes	Yes	Yes	Yes	Yes	Yes
Times Fes	Yes	Yes	Yes	Yes	Yes	Yes
Provinces Fes	Yes	Yes	Yes	Yes	Yes	Yes
Observations	378	378	378	378	378	378
R-squared	0.568	0.57	0.567	0.562	0.567	0.566
PANEL B: Infrastructures						
Dependent variables: Children under one year of age fully vaccinated						
$Moderate$	Class (total)	School (total)	Hospital (total)	Library (total)	Library books	Magazine office
Columns	(1)	(2)	(3)	(4)	(5)	(6)
GEOSS_t-1_	0.232***	0.147***	0.076***	0.081***	0.136***	0.027***
	(0.080)	(0.054)	(0.028)	(0.026)	(0.048)	(0.010)
GEOSS_t-1_ $\times \overline{Moderate }$	-0.026***	-0.024**	-0.071**	-0.028***	-0.023***	-0.013**
	(0.009)	(0.009)	(0.029)	(0.010)	(0.009)	(0.006)
Dynamics	Yes	Yes	Yes	Yes	Yes	Yes
Controls	Yes	Yes	Yes	Yes	Yes	Yes
Times Fes	Yes	Yes	Yes	Yes	Yes	Yes
Provinces Fes	Yes	Yes	Yes	Yes	Yes	Yes
Observations	378	378	378	378	378	378
R-squared	0.569	0.567	0.566	0.569	0.568	0.564

Source: Authors. The final data covers the years from 2014 to 2019. The values of the moderation variables are averaged throughout the study period, thus the characteristics regarding socio-economic status are relatively exogenous to the research model. Standard errors in parentheses *** *p* < 0.01, ** *p* < 0.05, * *p* < 0.1

These findings regarding heterogeneous effects are well-known compared to previous research [46, 85]. However, carefully considering the government’s next steps is crucial, given that prioritizing underdeveloped areas may reduce economic efficiency despite potentially yielding better health outcomes. Indeed, increasing expenditure in less developed regions resembles a “do less, achieve more” incentive, which many economic observers are concerned about. It should be noted that examining budget allocation from the central government to the 63 provinces/regions over the past two decades reveals a mechanism whereby Ho Chi Minh City and Hanoi, despite contributing the most to economic growth and budget, have to redistribute a significant portion of their budget (79% and 68% in 2023, respectively) (Vietnam National Assembly, [76]). Therefore, the interplay between equality and economic efficiency in this expenditure must be discussed carefully.

Finally, we verify the robustness of the model through estimation using two-stage least squares regression (column [1]) and GMM (columns [2]-[6]). Balance tax sources from the central government and targeted tax revenues are considered valid external instruments both theoretically and in technical tests, as presented at the end of the tables (e.g., AR(1), AR(2), test of over-restriction, and Weak identification test). The findings again confirm a consistent positive impact of GEOSS on the dependent variable. According to the rule of thumb, coefficients estimated in Table 6 using GMM are relatively consistent (0.008) with the estimated results (0.009), indicating either the study does not encounter serious endogeneity issues or the bias due to endogeneity is not severe.

**Table 6:** Estimation results with two-stage least square and two-system GMM

Dependent variable:	Children under one year of age fully vaccinated					
Estimators	XTIV2	GMM	GMM	GMM	GMM	GMM
Columns	(1)	(2)	(3)	(4)	(5)	(6)
Children fully vaccinated_t-1_	-0.071	0.068***	0.067***	0.019*	-0.069***	-0.055***
	(0.052)	(0.012)	(0.009)	(0.011)	(0.008)	(0.008)
GEOSS_t_	0.043*					
	(0.025)					
GEOSS_t-1_		0.002***	0.013***	0.011***	0.008***	0.008***
		(0.001)	(0.001)	(0.001)	(0.001)	(0.001)
Population on logarithm	0.006		-0.016***	-0.036***	-0.078***	-0.083***
	(0.032)		(0.001)	(0.002)	(0.007)	(0.009)
Literacy	-0.000			0.004***	0.006***	0.007***
	(0.002)			(0.000)	(0.001)	(0.001)
Private sector labor share	0.001					-0.001
	(0.001)					(0.001)
GRDP per capita on logarithm	-0.003				0.083***	0.086***
	(0.029)				(0.014)	(0.016)
Time Fes	Yes	Yes	Yes	Yes	Yes	Yes
Province Fes	Yes	Yes	Yes	Yes	Yes	Yes
Observations	378	378	378	378	378	378
R-squared	0.071					
Number of provinces/cities	63	63	63	63	63	63
External instruments	Balance tax sources from the central government and targeted tax revenues					
Internal instruments	No	1-year lagged	1-year lagged	1-year lagged	1-year lagged	1-year lagged
Underidentification test (Anderson LM statistic) (p-value)	0.0016					
Weak identification test (Cragg-Donald Wald F statistic)	9.977^a^					
Arellano-Bond test for AR(1)		0.001	0.001	0.001	0.001	0.001
Arellano-Bond test for AR(2)		0.541	0.658	0.706	0.622	0.587
Hansen test of overid. Restrictions (p-value)		0.209	0.158	0.310	0.421	0.363
Difference-in-Hansen tests of exogeneity		0.212	0.191	0.320	0.453	0.450

Source: Authors. The final data covers the years from 2014 to 2019. Standard errors are indicated in parentheses with significance levels: *** for *p* < 0.01, ** for *p* < 0.05, * for *p* < 0.1

^a^Critical values for the Stock-Yogo weak identification test: (a) 10% maximal IV size: 16.38 and (b) 15% maximal IV size: 8.96

Lastly, given our small panel data with short $T$ and large $N$, Appendix [3] employs estimates with feasible generalized least squares estimator (FGLS) and panel-corrected standard errors (PCSE) estimation to address cross-sectional dependence and heteroscedasticity [38, 39]. These coefficients are similar to pooled OLS but have adjusted variances to accommodate cross-sectional dependence and heteroscedasticity. The results confirm a consistently positive impact of $GEOSS$ on the vaccination rate, ranging from 0.007 to 0.009.

## Discussion and policy implication

Utilizing data from analysis units across 63 provinces in Vietnam from 2010 to 2019, the study shows a positive relationship between GEOSS and the vaccination rate of children under one year old and is in agreement with Francisco et al. [16]. We found that a 1% increase in GEOSS equates to a 0.8% increase in vaccination rates for children under 1 year old. This finding, when considered in the broader context of public investment in health, reaffirms the overall sentiment that increased investment yields better health returns [4, 50, 55]. Regarding the negative claim proposed in Kim and Moody [29], our findings do not directly disprove the claim, since the Vietnamese vaccination program is publicly funded, and differences due to income are negligible.

We also established a 1-year lag for local expenditure as an appropriate time to effect. A 1-year-lag favors the functional model proposed in Kutzin [30], whereas the Grossman model is harder to apply because there is no clear individual economic trade-off between consumption and government-sponsored vaccination. The estimated effect remains consistent after addressing endogeneity, cross-sectional dependence, and heteroscedasticity concerns. This suggests that governments should allocate budgetary resources towards vaccination operations. This initiative can be seen in action from Vietnam’s “Expanded Vaccination” program aimed at providing free immunization to children. Despite successes, vaccination shortages and budgetary constraints still persist [74], offering an immediate objective and necessity for additional targeted public health investments.

We provided a mechanism that links public investment, the number of healthcare workers, and vaccination rates. The size of the healthcare workforce is a positive determinant of health outcomes [3, 40] and GEOSS is positively correlated with the number of hospitals (see Appendix [3]). Thus, the three components can be linked through theoretical and empirical means. However, the literature on this matter is limited, we can only hypothesize and retroactively affirm the mechanism based on our data. There might be additional factors, such as administrative efficiency, that can affect the mechanism. Consequently, our mechanism at the moment resembles a framework for which future studies can further explore. Additionally, a key limitation of our proposed mechanism is the localization to Vietnam. Differences in healthcare laws across the world may require our theory to be modified. Further research is needed to arrive at a concrete conclusion.

The second research objective we intend to achieve is to examine the regional differences or heterogeneity in public spending. We discovered that funding impact is more potent in underdeveloped regions, where the proportion of females attending school is low and infrastructure is limited. The current study empirically confirms that allocating one dollar of the budget to underdeveloped areas yields better health outcomes than to already developed regions in Vietnam. Since healthcare and vaccination are considered services, our findings align with Cardona et al. [9]’s claim that investment into services is more effective in rural areas. This heterogeneous effect of funding on vaccination outcome has been documented in India in a study comparing high- (less developed) and non-high-focus (developed) states. However, Mohanty and Behera [46] reports higher coefficients in non-high-focus states on vaccination coverage compared to high-focus states. While the results seem contradictory, Mohanty and Behera [46] did not include similar control variables such as education (positively linked to vaccination and is higher in more developed areas) which may affect the reported values. Our conclusion suggests that governments should concentrate funding into less developed areas for higher returns in terms of vaccination coverage increases.

Yet, healthcare investment is an investment [12]. For every dollar commissioned to build a local health station or hire additional healthcare workers, that dollar is added to the local economy and can be used to boost local contractors, attract investors, and improve supporting infrastructure. This raises serious questions on the capability of less developed provinces to fully maximize the returns on taxpayer dollars from an economic standpoint, forming a so-called “trade-off” between economic and humanitarian benefits. Literature has documented this “trade-off” in health investment effectiveness between regions and populations [60]. Asamani et al. [5] mentioned studies that found evidence of reduced efficiency of healthcare investment in remote and rural areas due to underutilization. One can even argue that healthcare investment into more efficient regions generates higher returns that can be used to improve poorer regions, which would not be available had the government chosen poorer regions to initially invest.

This raises a dilemma about government interventions, particularly in a transitioning nation like Vietnam, regarding how adequate public funding for social endeavors should be when considering diverse objectives. In the scenario where the government pursues a strategy of social equity (investing more in underdeveloped regions), the evident consequence is that major cities like Ho Chi Minh City and Hanoi face shortages in public investment and a reduction in economic growth momentum [67]. Conversely, excessive favoritism towards economic objectives may compromise the legitimacy of a state adopting a socialist-oriented free-market economy system when equity is not ensured. Before the onset of the COVID-19 pandemic, we observed a shift in budget allocation strategies, with a focus on achieving a fair society. Major cities were seeing a decline in retained budget ratios, while additional budgets were being allocated to social endeavors in less developed provinces. However, the economic downturn triggered by the pandemic may significantly influence future decisions on local budget allocation mechanisms in Vietnam.

In conclusion, we found that public funding is a positive predictor of vaccination rates. However, heterogenous effects are observed in which the positive effect is larger in underdeveloped provinces compared to developed provinces. This creates a trade-off between economic and healthcare equity goals for policymakers to consider, especially in countries with unequal development distributions.

## Conclusion, limitations, and future directions

We examined the relationship between public funding, proxied by government expenditure on social securities, and vaccination rate in children under one-year-old. A positive relationship between public funding and children's vaccination rate was found, which was consistent after robustness checks. In addition, heterogeneity in funding effectiveness was also discovered, agreeing with published literature that health investment in rural, low infrastructure and institutional quality areas is more effective than in urban, developed areas. Thus, policymakers should aim to invest more in healthcare and services if vaccination rates are to be improved and target rural areas to see maximum improvement returns. We also proposed a mechanism in which increased funding increases the number of medical stations and healthcare workers, thereby boosting the vaccination rate.

However, the study faces limitations in that the healthcare system and factors might be unique to Vietnam, a socialist country with a strong public healthcare commitment. The same model may be applied in other countries but yield different results. Future research should focus on confirming or establishing a mechanism for vaccination rate increase, as well as adapting the study’s model to study different healthcare systems around the world.

## Data Availability

No datasets were generated or analysed during the current study.

## Appendix 1

**Table:** The CombeFive vaccine (2019) to vaccination rates in Vietnam

Dependent variable	Children under one year of age fully vaccinated			
Columns	(1)	(2)	(3)	(4)
Dummy (=1 if the year is 2019, =0 if otherwise)	-0.012**	-0.012**	-0.022***	-0.023***
	(0.006)	(0.005)	(0.005)	(0.004)
Children fully vaccinated_t-1_			0.176***	-0.011
			(0.049)	(0.031)
Province Fes	No	Yes	No	Yes
Observations	504	504	378	378
R-squared	0.008	0.244	0.137	0.484

Source: Authors. The samples in columns [1] and [2] cover the years 2010 and 2013-2019, resulting in 504 observations. The samples in columns [3] and [4] cover the years 2014-2019, with 378 observations. Robust standard errors in parentheses ****p* < 0.01, ** for *p* < 0.05, * for *p* < 0.1

## Appendix 2

**Table:** Results of Table 5

PANEL A: Female students & Insitutional quality						
Dependent variables	Children under one year of age fully vaccinated					
Moderate	Female student on logarithm	Preprimary female student on logarithm	Secondary female student on logarithm	High school female student on logarithm	Business support services	Law & Order
Estimators	FEM	FEM	FEM	FEM	FEM	FEM
Columns	(1)	(2)	(3)	(4)	(5)	(6)
Children fully vaccinated_t-1_	-0.047	-0.046	-0.048	-0.046	-0.047	-0.029
	(0.043)	(0.043)	(0.043)	(0.043)	(0.043)	(0.043)
GEOSS_t-1_	0.258***	0.283***	0.222***	0.118*	0.127***	0.118**
	(0.093)	(0.093)	(0.084)	(0.061)	(0.046)	(0.046)
GEOSS_t-1_ $\times \overline{Moderate }$	-0.022***	-0.026***	-0.021**	-0.011*	-0.022***	-0.019**
	(0.008)	(0.009)	(0.008)	(0.006)	(0.009)	(0.008)
Population on logarithm	0.021	0.020	0.021	0.021	0.021	0.020
	(0.028)	(0.027)	(0.028)	(0.028)	(0.028)	(0.028)
Literacy	0.001	0.001	0.001	0.001	0.000	0.001
	(0.002)	(0.002)	(0.002)	(0.002)	(0.002)	(0.002)
Private sector labor share	0.001	0.001	0.001	0.001	0.001*	0.001
	(0.001)	(0.001)	(0.001)	(0.001)	(0.001)	(0.001)
GRDP per capita on logarithm	0.009	0.008	0.009	0.009	0.009	0.010
	(0.026)	(0.026)	(0.026)	(0.026)	(0.026)	(0.026)
Times Fes	Yes	Yes	Yes	Yes	Yes	Yes
Provinces Fes	Yes	Yes	Yes	Yes	Yes	Yes
Observations	378	378	378	378	378	378
R-squared	0.568	0.570	0.567	0.562	0.567	0.566
PANEL B: Infrastructures						
Dependent variables	Children under one year of age fully vaccinated					
Moderate	Class numbers on logarithm	School numbers on logarithm	Hospital numbers on logarithm	Library number on logarithm	Library book numbers on logarithm	Magazine office on logarithm
Estimators	FEM	FEM	FEM	FEM	FEM	FEM
Columns	(1)	(2)	(3)	(4)	(5)	(6)
Children fully vaccinated_t-1_	-0.048	-0.050	-0.046	-0.054	-0.048	-0.043
	(0.043)	(0.043)	(0.043)	(0.043)	(0.043)	(0.043)
GEOSS_t-1_	0.232***	0.147***	0.076***	0.081***	0.136***	0.027***
	(0.080)	(0.054)	(0.028)	(0.026)	(0.048)	(0.010)
GEOSS_t-1_ $\times \overline{Moderate }$	-0.026***	-0.024**	-0.071**	-0.028***	-0.023***	-0.013**
	(0.009)	(0.009)	(0.029)	(0.010)	(0.009)	(0.006)
Population on logarithm	0.021	0.024	0.030	0.024	0.013	0.019
	(0.027)	(0.028)	(0.028)	(0.028)	(0.027)	(0.028)
Literacy	0.001	0.000	0.000	0.000	0.001	0.000
	(0.002)	(0.002)	(0.002)	(0.002)	(0.002)	(0.002)
Private sector labor share	0.001	0.001	0.001*	0.001	0.001	0.001
	(0.001)	(0.001)	(0.001)	(0.001)	(0.001)	(0.001)
GRDP per capita on logarithm	0.009	0.013	0.018	0.011	0.000	0.006
	(0.026)	(0.026)	(0.027)	(0.026)	(0.026)	(0.026)
Times Fes	Yes	Yes	Yes	Yes	Yes	Yes
Provinces Fes	Yes	Yes	Yes	Yes	Yes	Yes
Observations	378	378	378	378	378	378
R-squared	0.569	0.567	0.566	0.569	0.568	0.564

Source: Authors. The final data covers the years from 2014 to 2019. Standard errors are indicated in parentheses with significance levels: *** for *p* < 0.01, ** for *p* < 0.05, * for *p* < 0.1

## Appendix 3

**Table:** Regression results with different estimators

Dependent variable:	Children under one year of age fully vaccinated			
Estimators	GLS	GLS	PCSE	PCSE
Columns	(1)	(2)	(3)	(4)
Children fully vaccinated_t-1_	0.167***	-0.037	0.167	-0.037
	(0.041)	(0.039)	(0.104)	(0.084)
Government expenditure on social securities_t-1_	0.007***	0.009**	0.007**	0.009***
	(0.003)	(0.004)	(0.003)	(0.004)
Population on logarithm	-0.014***	0.016	-0.014***	0.016
	(0.003)	(0.025)	(0.005)	(0.027)
Literacy	0.001***	0.000	0.001***	0.000
	(0.000)	(0.001)	(0.000)	(0.002)
Private sector labor share	0.000**	0.001	0.000***	0.001
	(0.000)	(0.000)	(0.000)	(0.001)
GRDP per capita on logarithm	-0.000	0.005	-0.000	0.005
	(0.003)	(0.023)	(0.001)	(0.026)
Time Fes	Yes	Yes	Yes	Yes
Province Fes	No	Yes	No	Yes
Observations	378	378	378	378
R-squared			0.312	0.558
Number of provinces/cities	63	63	63	63

Source: Authors. The final data covers the years from 2014 to 2019. Standard errors are indicated in parentheses with significance levels: *** for *p* < 0.01, ** for *p* < 0.05, * for *p* < 0.1
